# Plasma chemokines as immune biomarkers for diagnosis of pediatric tuberculosis

**DOI:** 10.1186/s12879-021-06749-6

**Published:** 2021-10-11

**Authors:** Nathella Pavan Kumar, Syed Hissar, Kannan Thiruvengadam, Velayuthum V. Banurekha, Sarath Balaji, S. Elilarasi, N. S. Gomathi, J. Ganesh, M. A. Aravind, Dhanaraj Baskaran, Srikanth Tripathy, Soumya Swaminathan, Subash Babu

**Affiliations:** 1 International Center for Excellence in Research, National Institute for Research in Tuberculosis , Chennai, India; 2 ICMR-National Institute for Research in Tuberculosis, Chennai, India; 3 Institute of Child Health and Hospital for Children, Chennai, India; 4 Government Stanley Medical College and Hospital, Chennai, India; 5 World Health Organisation, Geneva, Switzerland; 6 LPD, NIAID, NIH, Bethesda, MD USA

**Keywords:** Pediatric tuberculosis, Chemokines, Biomarkers

## Abstract

**Background:**

Diagnosing tuberculosis (TB) in children is challenging due to paucibacillary disease, and lack of ability for microbiologic confirmation. Hence, we measured the plasma chemokines as biomarkers for diagnosis of pediatric tuberculosis.

**Methods:**

We conducted a prospective case control study using children with confirmed, unconfirmed and unlikely TB. Multiplex assay was performed to examine the plasma CC and CXC levels of chemokines.

**Results:**

Baseline levels of CCL1, CCL3, CXCL1, CXCL2 and CXCL10 were significantly higher in active TB (confirmed TB and unconfirmed TB) in comparison to unlikely TB children. Receiver operating characteristics curve analysis revealed that CCL1, CXCL1 and CXCL10 could act as biomarkers distinguishing confirmed or unconfirmed TB from unlikely TB with the sensitivity and specificity of more than 80%. In addition, combiROC exhibited more than 90% sensitivity and specificity in distinguishing confirmed and unconfirmed TB from unlikely TB. Finally, classification and regression tree models also offered more than 90% sensitivity and specificity for CCL1 with a cutoff value of 28 pg/ml, which clearly classify active TB from unlikely TB. The levels of CCL1, CXCL1, CXCL2 and CXCL10 exhibited a significant reduction following anti-TB treatment.

**Conclusion:**

Thus, a baseline chemokine signature of CCL1/CXCL1/CXCL10 could serve as an accurate biomarker for the diagnosis of pediatric tuberculosis.

**Supplementary Information:**

The online version contains supplementary material available at 10.1186/s12879-021-06749-6.

## Background

Diagnosis of active tuberculosis (TB) in children provides a key challenge, because most of the clinical symptoms of TB in children are non-specific with signs and symptoms that resemble common pediatric illnesses, including pneumonia and malnutrition and therefore clinical diagnosis is unreliable [1]. Using respiratory specimens for the diagnosis of bacterial confirmation in young children is difficult because they are unable to expectorate sputum. In addition, the diagnostic yields in the sputum sample are not high due to the paucibacillary nature of the disease [2, 3]. Thus, a novel, non-sputum-based point-of-care (POC) diagnostic tool that could give an early and precise diagnosis of TB disease in children is urgently required. A recent WHO report states the need for a rapid biomarker based non-sputum-dependent diagnostic test for the diagnosis of pulmonary TB [4]. Children are clinically crucial populations with greater susceptibility to tuberculosis, and mechanisms of protection are still unclear [5]. WHO reports that among the 10 million new cases of active TB each year, approximately 10% happen in children less than 15 years of age [6]. *Mycobacterium tuberculosis *(*M.tb*) exposed children acquire infection, which is described as immunological evidence of sensitisation to *M.tb*, and some of these children may develop TB disease with signs and symptoms with microbiological evidence of disease [7]

It has also been reported in a few studies that IP-10 detection could be useful for diagnosing active TB and LTBI in children [8] and even in the adult population, IP-10 and MCP-2 [9] can be used as biomarkers for tuberculosis. In addition, some studies with smaller sample size have shown that IP-10, IFN-γ and IL-2 can be used as a diagnostic tool for pediatric tuberculosis [10]. However, most of these studies used only the antigen specific chemokine or cytokine response for diagnosis.

Better-quality methods for diagnosing pediatric TB are thus immediately needed and immunological biomarker assays can bring light to this problem. Chemokines are chemoattractant molecules that control immunological responses and have been widely considered for their utility as diagnostic biomarkers of adult TB [11]. Results of our previous published studies in adults showed that plasma chemokines are significantly elevated in an active TB group and can significantly discriminate active TB from latent TB or healthy controls [12]. In this proof-of-concept study, we aimed to determine the plasma concentrations of CC (CCL1, CCL2, CCL3, CCL4 and CCL11) and CXC (CXCL1 CXCL2, CXCL9, CXCL10 and CXCL11) chemokines as markers capable of discriminating among children who were microbiology positive (confirmed TB) or those negative but clinically diagnosed and treated (unconfirmed TB) compared with unlikely TB children in a prospectively recruited cohort for the study.

## Methods

### Study population and procedures

We conducted a prospective case control study using children with confirmed, unconfirmed and unlikely TB. All admitted and out patients in the pediatric wards of Institute of Child Health and Hospital for Children, Chennai and Government Stanley Medical College and Hospital, Chennai, were screened. Children aged less than 15 years of age with symptoms suggestive of Intrathoracic tuberculosis were enrolled between February 2016 and March 2018. Of the 195 children screened, 167 children were recruited which includes 44 children who were microbiology positive (confirmed TB) for *M.tb,* 47 children who were microbiology negative (unconfirmed TB), 76 children with other respiratory ailments with tuberculin skin Test (TST) positive or negative as unlikely TB controls. Two sputum samples were collected from each child on two consecutive days and gastric aspirates were collected for children aged < 5. All children in the study population underwent sputum smear and culture (or Xpert MTB/RIF) and Tuberculin Skin Test (TST).

Definition of clinical groups [13]

*Confirmed TB:* Microbiologically positive for *M.tb*. Positive for Xpert MTB/RIF or smear or culture for *M.tb.*

*Unconfirmed TB*: Clinical features suggestive of TB or abnormal chest X-ray or history of household TB contact or response to anti-tuberculosis treatment (ATT) [at least two of the criteria were met].

*Unlikely TB:* Children who had a differential diagnosis apart from TB and who were either negative or positive for TST results were considered as controls.

Unlikely TB children were children who were suspected of having TB but clinically diagnosed as having other respiratory illness like COPD, viral pneumonia, bacterial pneumonia or Asthma/wheeze, empirically treated with antibiotics/antivirals/anti asthmatic medication and followed up for a period of 8 weeks for resolutions of symptoms, resolution of chest x-ray abnormalities and confirmed as TB culture negative.

At the time of enrolment, all active TB cases (Confirmed and Unconfirmed TB together) had no record of prior TB disease or ATT. A positive TST result was defined as an induration at the site of tuberculin inoculation of at least 10 mm in diameter. Plasma samples were collected from all of the above children in a consecutive sampling method. From the confirmed TB and unconfirmed TB children blood samples were collected before starting the anti-tuberculosis treatment. All the blood samples were collected in sodium heparin tubes. Collected blood samples were transported within 2 h to the Immunology lab for processing and further plasma samples were stored in − 80 °C freezer. All children were HIV negative. All confirmed and unconfirmed TB children were administered ATT for 6 months. At 6 months following ATT initiation, fresh plasma samples were obtained from a subset of confirmed TB (n = 24) and unconfirmed TB (n = 23) children. All children with active TB were culture negative and symptom free at the end of ATT. The demographic and epidemiological data have been previously reported [14].

### Multiplex chemokine assay

Plasma levels of CC (CCL1, CCL2, CCL3, CCL4 and CCL11) and CXC (CXCL1 CXCL2, CXCL9, CXCL10 and CXCL11) chemokines were measured using the Luminex Magpix multiplex cytokine assay system (Bio-Rad, Hercules, CA, USA). Luminex Human Chemokine Magnetic Assay kit (R & D systems, USA) was used to measure the chemokine levels. All the assays were performed in the stored samples. The lowest detection limits were as follows: CCL1, 4.5 pg/mL; CCL2, 31.8 pg/mL; CCL3, 30.9 pg/mL; CCL4, 133.4 pg/mL; CCL11, 21.6 pg/mL; CXCL1, 49.2 pg/mL; CXCL2, 49.2 pg/mL; CXCL9, 600 pg/mL, CXCL10, 2.8 pg/mL and CXCL11, 21.6 pg/mL. N = 44 samples were analysed per batch in duplicates, and lab personal were blinded to the clinical groups. All the assays were performed according to the manufacturer’s instructions.

### Statistical analysis

Geometric means (GM) were used for measurements of central tendency. Statistically significant differences between confirmed TB, unconfirmed TB and unlikely TB children were analysed using the Kruskal–Wallis test with Dunn’s multiple comparisons. Wilcoxon signed rank test was used to compare cytokine concentrations before and after ATT. Receiver Operator Characteristics (ROC) curves were used to determine accuracy of each candidate chemokine immune biomarker to distinguish confirmed TB, unconfirmed TB and unlikely TB. Analyses were performed using Graph-Pad PRISM Version 9.0 (GraphPad Software, CA, USA). Values below the detection limits were handled using the least squared (ordinary) fit method to imputed the quantitative value and as such included in graphs and for the statistical analysis [15]. Computation and selection of optimal biomarker combinations by integrative ROC were analysed using a freely available web application (http://CombiROC.eu) CombiROC v.1.2. [16]. Classification and regression trees (CART) model were employed to identify the cut-off values for the biomarkers which separate the active TB children from those with unlikely TB. The analysis was done using the R (R Foundation for Statistical Computing, Vienna, Austria) software.

## Results

### Participant categorisation

**Fig. 1:**
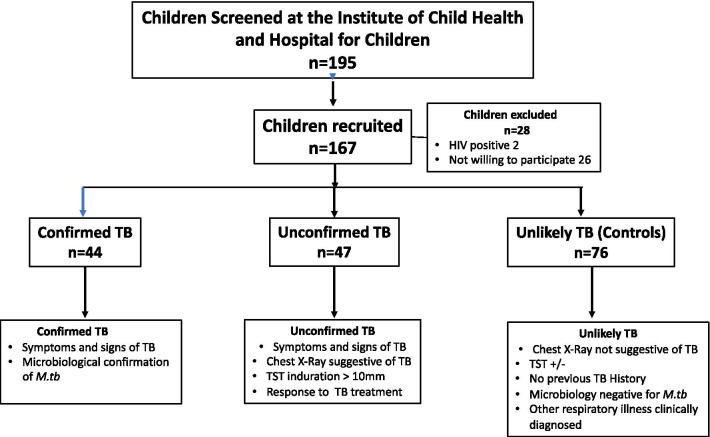
Outline of participant categorisation. In the study cohort (*N* = 167), plasma samples were collected from children who were microbiology positive (confirmed TB) or those negative (unconfirmed TB) for *M.tb* but with symptoms that suggested tuberculosis at baseline and at the end of ATT. Children with other respiratory ailments as unlikely TB were also studied at baseline as controls

The recruitment algorithm for the children is shown in Fig. 1. Of the 195 children screened, 167 were recruited. The demographics of the children are shown in Table 1. We had 20 children below 5 years, 8 children between 5 and 10 and 16 children between 10 and 12 years in the confirmed TB group; and 21 children below 5 years, 6 children between 5 and 10 years and 20 children between 10 and 12 years in the unconfirmed TB group. All children in the confirmed and unconfirmed TB group were TST positive.

**Table 1:** Study demographics

Demographic characteristic	Confirmed TB	Unconfirmed TB	Unlikely TB
Number of subjects recruited	44	47	76
Gender (male / female)	16/28	28/19	36/40
Median age (range) (in years)	7 (1–13)	8 (1–12)	6 (1–14)
Bacterial burden:	11/33/0	0/0/47	–
High burden/ low burden / no burden			
Tuberculin skin test: (positive/negative)	44/0	35/12	45/31

### Plasma levels of chemokines are elevated in children with active TB disease

**Fig. 2:**
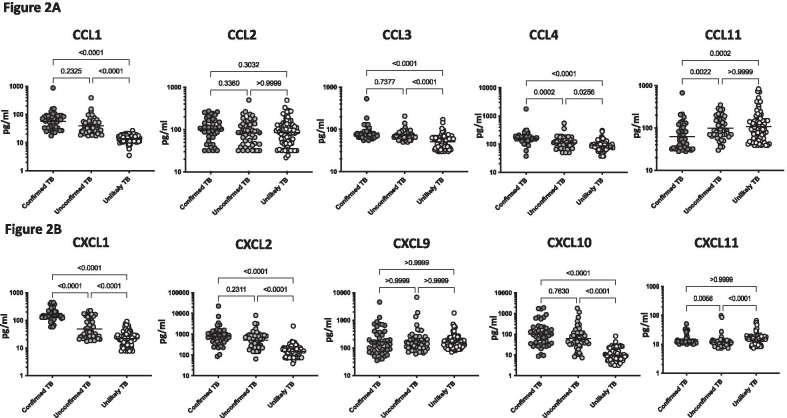
Elevated circulating levels of CC and CXC chemokines in children with active TB disease. **A** The plasma levels of CCL1, CCL2, CCL3, CCL4, CCL11 and **B** CXCL1, CXCL2, CXCL9, CXCL10 and CXCL11 were measured in confirmed TB (n = 44), unconfirmed TB (n = 47) and unlikely TB (n = 76) individuals at baseline. The data are represented as scatter plots with each circle representing a single individual. P values were calculated using the Kruskal–Wallis test with Dunn’s post-hoc for multiple comparisons

To determine the levels of plasma chemokines in children with active TB disease and with no TB disease (but with other respiratory illness), we measured the plasma levels of CC (CCL1, CCL2, CCL3, CCL4 and CCL11) and CXC (CXCL1 CXCL2, CXCL9, CXCL10 and CXCL11) in children with confirmed TB (n = 44), unconfirmed TB (n = 47) and unlikely TB (n = 76) (Fig. 2). As shown in Fig. 2, the plasma levels of CC and CXC chemokines—CCL1 (Geometric Mean (GM) of 57.2 pg/ml in confirmed TB vs 39.14 pg/ml in unconfirmed TB and 14.01 pg/ml in Unlikely TB), CCL3 (GM of 81.7 pg/ml in confirmed TB vs 73.4 pg/ml in unconfirmed TB and 51 pg/ml in Unlikely TB), CXCL1 (GM of 157.6 pg/ml in confirmed TB vs 48.2 pg/ml in unconfirmed TB and 21.5 pg/ml in Unlikely TB), CXCL2 (GM of 809.1 pg/ml in confirmed TB vs 514.4 pg/ml in unconfirmed TB and 147.6 pg/ml in Unlikely TB) and CXCL10 (GM of 102.7 pg/ml in confirmed TB vs 62.8 pg/ml in unconfirmed TB and 9.8 pg/ml in Unlikely TB) were significantly higher in children with confirmed and unconfirmed TB compared to unlikely TB children.

### Plasma chemokines can robustly distinguish confirmed and unconfirmed TB from unlikely TB

**Fig. 3:**
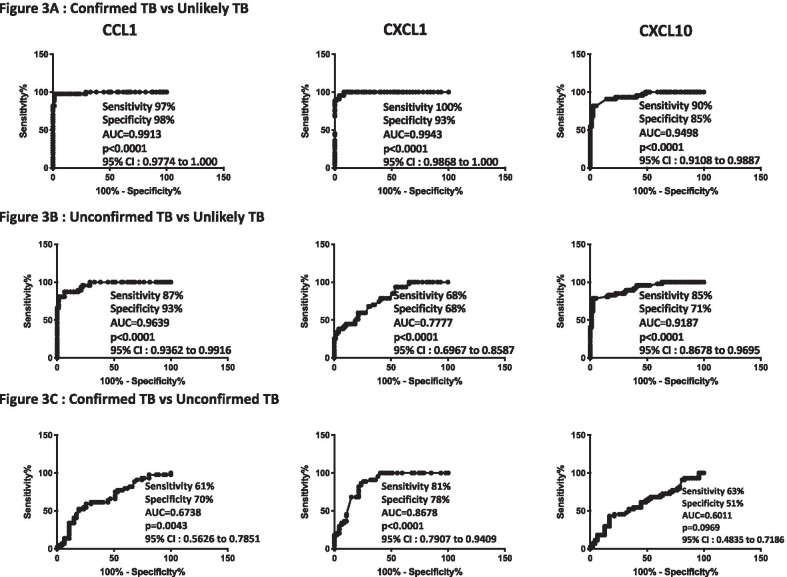
ROC analysis to estimate the discriminatory power of CC and CXC chemokines in children with active TB disease and unlikely TB. ROC analysis to estimate the sensitivity, specificity and AUC was performed using CCL1, CXCL1 and CXCL10 to estimate the capacity of these factors to distinguish individuals with (**A**) confirmed TB vs. unlikely TB (**B**) Unconfirmed TB vs. unlikely TB (**C**) confirmed TB vs. unconfirmed TB. *ROC  *receiver operator characteristics

To determine the discriminatory power of plasma CC and CXC chemokines in distinguishing children with confirmed and unconfirmed TB from unlikely TB, we performed ROC analysis of CCL1, CXCL1 and CXCL10 in confirmed and unconfirmed TB vs. unlikely TB children and confirmed vs unconfirmed TB (Fig. 3). As shown in Fig. 3A, CCL1 (AUC 0.9913, sensitivity 97%, specificity 98%), CXCL1 (AUC 0.9943, sensitivity 100%, specificity 93%), CXCL10 (AUC 0.9498, sensitivity 90%, specificity 85%) exhibited significant discriminatory power with high AUC values, sensitivity and specificity in discriminating confirmed TB from unlikely TB children. Similarly, as shown in Fig. 3B, CCL1 (AUC 0.9639, sensitivity 87%, specificity 93%), CXCL1 (AUC 0.777, sensitivity 68%, specificity 68%), CXCL10 (AUC 0.9187, sensitivity 85%, specificity 71%), exhibited significant discriminatory power with high AUC values, sensitivity and specificity in discriminating unconfirmed TB from unlikely TB children. Finally, as shown in Fig. 3C, CCL1 (AUC 0.6738, sensitivity 61%, specificity 70%), CXCL1 (AUC 0.8678, sensitivity 81%, specificity 78%) exhibited a significant difference between confirmed TB vs unconfirmed TB children but with a low sensitivity and specificity and CXCL10 (AUC 0.6011, sensitivity 63%, specificity 51%) exhibited no significant discriminatory power between confirmed TB vs unconfirmed TB children. Thus, plasma chemokines exhibit the potential to serve as biomarkers to distinguish both confirmed and unconfirmed TB disease from unlikely TB children.

### A plasma signature of two or three chemokines is an accurate biomarker discriminating confirmed or unconfirmed TB disease from unlikely TB

**Fig. 4:**
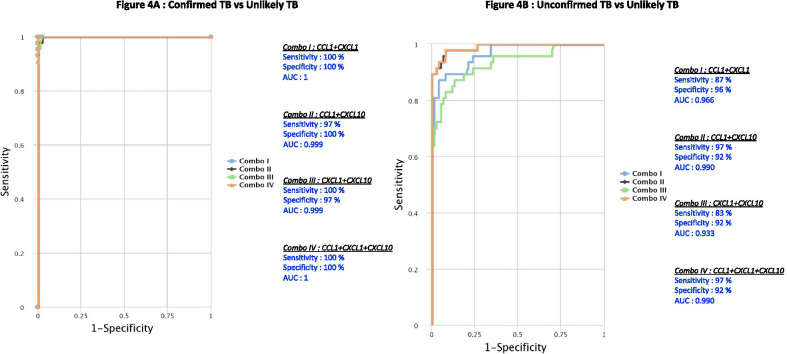
Identification of biomarkers showing the strongest association using combination of chemokine biomarkers in active TB disease. CombiROC model analysis shows the chemokines that exhibited the highest accuracy in discriminating confirmed TB and unconfirmed TB disease from unlikely TB. ROC curves for comparing multiple markers and their combinations between confirmed TB and unconfirmed TB versus unlikely TB (**A**) Confirmed TB vs. unlikely TB (**B**) unconfirmed TB vs. unlikely TB are shown

A combinatorial analysis of multiple immune biomarkers was carried out to define the POC diagnostic accuracy of ideal marker combinations of the tested circulating plasma chemokines using the CombiROC method. This aims to examine the best combinations of biomarkers through a combined analysis of ROC curves, considering the sensitivity and specificity of all possible immune markers [17]. The examined chemokines as multiple marker signatures were evaluated in different combinations using CombiROC and the best combinations with the highest AUC, sensitivity and specificity were selected (Fig. 4). As shown in Fig. 4A, the dual combination of chemokines CCL1/CXCL1 (AUC 1, sensitivity 100%, specificity 100%) CCL1/CXCL10 (AUC 0.999, sensitivity 97%, specificity 100%), CXCL1/CXCL10 (AUC 0.999, sensitivity 100%, specificity 97%) and triple combination of chemokines CCL1/CXCL1/CXCL10 (AUC 1, sensitivity 100%, specificity 100%) showed significant discriminatory power with high AUC, sensitivity and specificity in discriminating confirmed TB from unlikely TB children. Similarly, as shown in Fig. 4B, the dual combination of chemokines CCL1/CXCL1 (AUC 0.966, sensitivity 87%, specificity 96%) CCL1/CXCL10 (AUC 0.990, sensitivity 97%, specificity 92%), CXCL1/CXCL10 (AUC 0.933, sensitivity 83%, specificity 92%) and triple combination of chemokines CCL1/CXCL1/CXCL10 (AUC 0.990, sensitivity 97%, specificity 92%) showed significant discriminatory power with high AUC, sensitivity and specificity in discriminating unconfirmed TB from unlikely TB children. Overall, we found that a biomarker signature of two or three chemokines exhibited excellent predictive performance in both confirmed and unconfirmed TB.

### Biomarkers discriminating active TB disease from unlikely TB

**Fig. 5:**
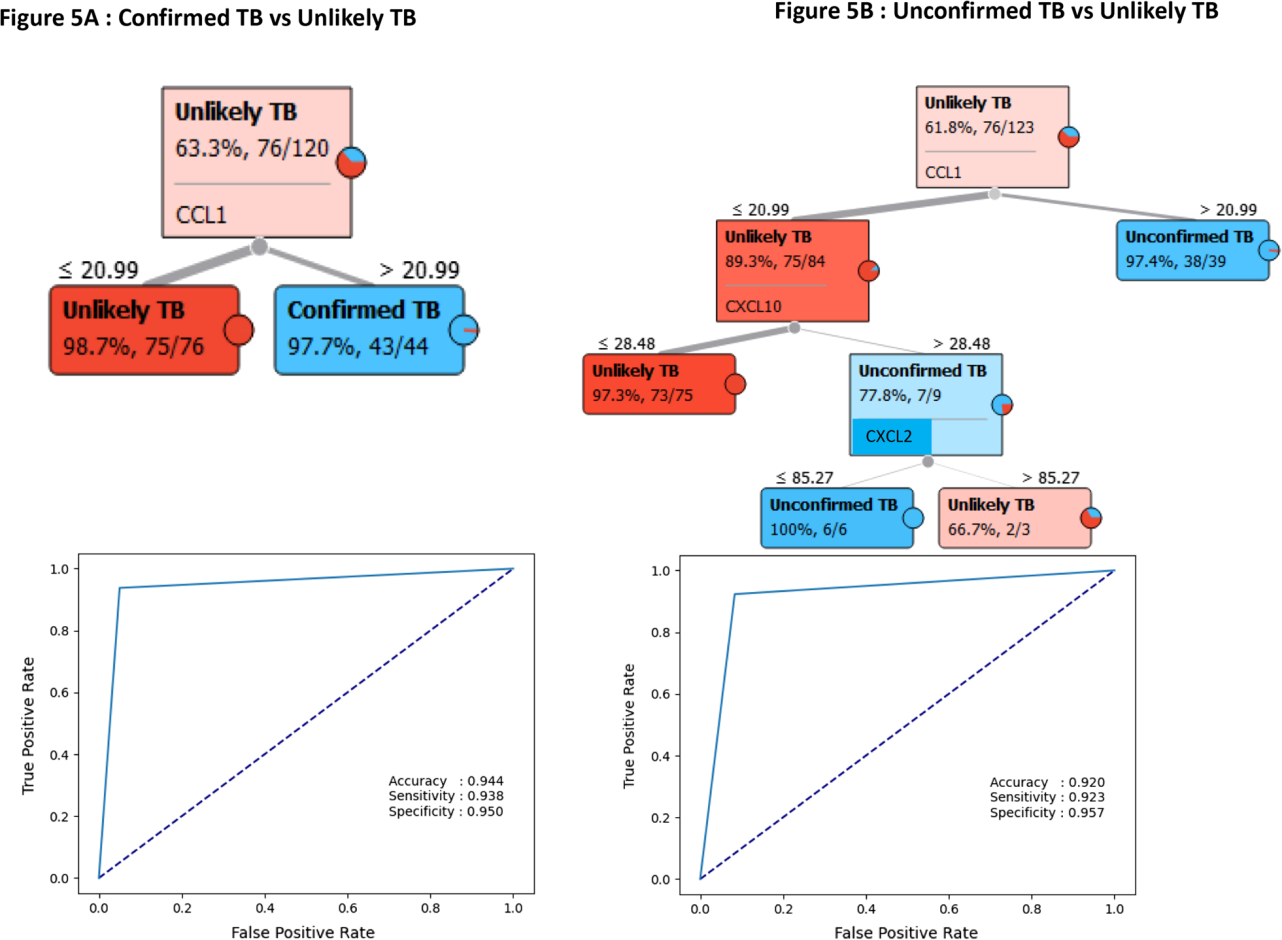
Identification of biomarkers showing the strongest associations with active TB disease. CART model analysis shows the chemokines that exhibited the highest accuracy in discriminating (**A**) Confirmed TB vs unlikely and ROC curves were employed to quantify the accuracy of single or combined biomarkers. **B** Unconfirmed TB vs unlikely TB and ROC curves were employed to quantify the accuracy of single or combined biomarkers. Cut-off value of CCL1is determined by this model

Classification and regression trees (CART) models were employed to identify the cut-off for the biomarkers which separate the active TB disease from those with unlikely TB children. We have used the data from all the markers for the tree construction and then we selected the most relevant biomarker that classifies the group more accurately (Fig. 5). As shown in Fig. 5A, CCL1 with a cut-off value of 20.99 pg/ml was classified by the CART model, which demonstrated that the model was able to discriminate samples with the AUC 0.944, sensitivity 93%, specificity 95% in confirmed TB vs unlikely TB. As shown in Fig. 5B, CCL1 with cut-off value of 20.99 pg/ml, CXCL10 cut-off value of 28.48 pg/ml and CXCL2 cut-off value of 85.27 pg/ml were classified by the CART model which determined that the model was able to discriminate samples with the AUC 0.920, sensitivity 92%, specificity 95% in unconfirmed TB vs unlikely TB. This CART analysis was able to demonstrate that CCL1 with a cut off value of 20.99 pg/ml is a sensitive diagnostic immune biomarker for diagnosis of active TB in children.

### Plasma chemokines levels are significantly diminished following ATT

**Fig. 6:**
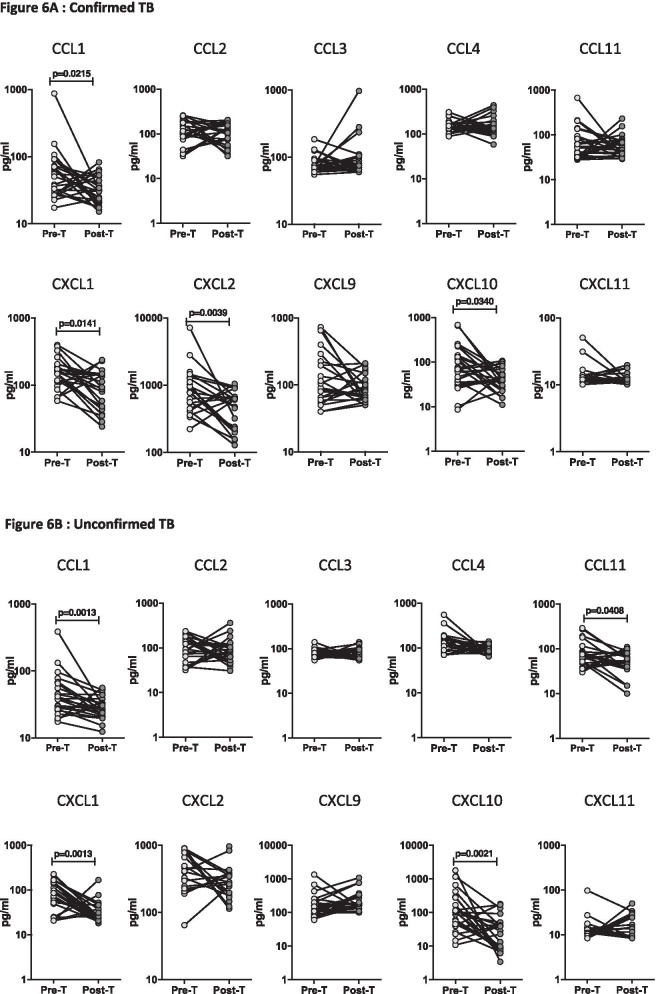
Diminished plasma levels of chemokines at the end of standard anti-tuberculosis (TB) therapy in children with active TB disease. The plasma levels of CC and CXC chemokines at baseline (pre-T) and at 6 months of anti-TB treatment (post-T) in a subset of (**A**) Confirmed TB (n = 24) and (**B**) Unconfirmed TB (n = 23). The data are presented as line graphs with each line representing a single individual

To elucidate whether the increased levels of chemokines are directly related with TB disease, we measured the levels of these chemokines in a subgroup of children with confirmed TB (n = 24) and unconfirmed TB (n = 23) before and after a standard course of ATT (Pre-Treatment (pre-T) versus Post-Treatment (post-T). As shown in Fig. 6A, in children with confirmed TB at the end of ATT, the plasma levels of CCL1 (GM of 54.1 pg/ml in pre-T vs. 31.1 pg/ml in post-T), CXCL1 (GM of 147.8 pg/ml in pre-T vs. 88.7 pg/ml in post-T), CXCL2 (GM of 768.2 pg/ml in pre-T vs. 370.1 pg/ml in post-T), CXCL10 (GM of 75.9 pg/ml in pre-T vs. 40.5 pg/ml in post-T) were significantly decreased in comparison to pre-treatment levels. Likewise, as shown in Fig. 6B, in children with unconfirmed TB at the end of ATT, the plasma levels of CCL1 (GM of 42.5 pg/ml in pre-T vs. 27.7 pg/ml in post-T), CCL11 (GM of 13 pg/ml in pre-T vs. 14.1 pg/ml in post-T), CXCL1 (GM of 77.7 pg/ml in pre-T vs. 30.4 pg/ml in post-T) and CXCL10 (GM of 105 pg/ml in pre-T vs. 21.9 pg/ml in post-T) were also significantly diminished in comparison to pre-treatment levels. Next, we also wanted to assess whether the chemokines levels at the end of ATT in the confirmed TB group were similar to that of the unlikely TB group at baseline. As shown in Additional file 1: Fig. S1, children with confirmed TB at the end of ATT, exhibited levels of CCL1, CCL3, CCL4, CXCL1, CXCL2 and CXCL10, that were significantly increased while the levels of CCL11 and CXCL9 were significantly decreased in comparison to unlikely TB group.

## Discussion

Pediatric populations are at a higher risk to progress to active TB disease following primary infection compared to adults [18]. Furthermore, extrapulmonary TB or disseminated forms of the TB disease are more common in young children [19]. Novel diagnostic biomarkers are still needed for the diagnosis of pediatric TB population and existing immune biomarker research for this target thus far has yielded partial success [1, 20, 21]. Despite the desperate need for better diagnosis of TB in children, only limited knowledge is currently available for understanding the mechanisms responsible for the disease severity during pediatric tuberculosis [22, 23]. Previously published studies on chemokines have prominently contributed to the understanding of TB pathogenesis and also findings from adult TB reported that chemokines might differentiate active TB from latent TB and healthy controls [11]. However, most of the studies in this field were performed in people more than 15 years old. Hence, in this study we sought to fill this knowledge gap by examining plasma chemokines in children with confirmed TB, unconfirmed TB and unlikely TB. Our results add to the prevailing knowledge in the field by validating that plasma immune biomarkers like CCL1, CCL3, CXCL1, CXCL2 and CXCL10 can strikingly distinguish confirmed TB or unconfirmed TB from unlikely TB controls children in a highly endemic region. These blood-based immune biomarkers could perhaps be used as targets for a POC test without dependency on a complex laboratory arrangement.

Overall, results from our study exhibited that CC chemokines—CCL1 and CCL3 as well as the CXC chemokines—CXCL1, CXCL2 and CXCL10 are present at significantly higher concentrations in the plasma of active TB children in comparison to unlikely TB children but with other respiratory ailments. Interestingly no difference in CXCL9 was observed, which is different from what was reported previously in adults [12] and this could be attributable to differences in chemokine levels of adults and children. In addition, the plasma concentration of CXCL9 and CXCL10 were lower compare to other published studies due to the fact most of these studies are in adults [24].

For the recruitment of cells into the *M.tb*-infected lung, chemokines play a dominant role, which in turn leads to control of *M.tb* infection [25]. However, under certain conditions of lung damage and inflammation, chemokines drive the pathogenesis leading to severe TB disease [25, 26]. Previous studies, including our own in adult TB patients, have reported results regarding the diagnostic potential of chemokine biomarkers to identify TB disease from those who are uninfected in a population with endemic TB setting [12]. Previous studies suggest that CXCL10 (IP-10) is a promising immune biomarker for diagnosis of either latent TB or active TB disease [27]. To support our existing data, a recent study in children with pediatric TB reported that CXCL10 (IP-10) showed a significant difference between the TB children (confirmed TB and unconfirmed TB) when compared with unlikely TB children [28]. In addition, the authors of the study also reported that the combination of IP-10 with IL-7 and IL-1ra markers showed an average sensitivity of 70% and specificity of 75% [29], whereas in our study, the combination of CXCL10 with other chemokines such as CCL1 and CXCL1 showed 100% sensitivity and specificity in confirmed TB and sensitivity of 97% and specificity of 92% in unconfirmed TB in comparison to unlikely TB. Further, in order to test the diagnostic accuracy of these markers, ROC curve analysis was applied in the study population. The results show that CCL1, CXCL1 and CXCL10 could clearly discriminate confirmed TB children from unlikely TB controls with more than 90% sensitivity, specificity and good AUC, whereas CCL1 and CXCL10 alone showed a good sensitivity, specificity and AUC between unconfirmed TB and unlikely TB children. The results of this study further confirm that our diagnostic chemokine biomarkers perform well in this population with a greater background prevalence of LTB at the population level.

We have developed a three-immune biosignature of chemokines which could efficiently discriminate confirmed and unconfirmed TB from unlikely TB with good accuracy. Our current results report that three chemokine immune biomarkers (CCL1, CXCL10 and CXCL1) had the ability to discriminate between active TB disease and non-TB disease in children with high levels of sensitivity and specificity. Among these three chemokines, results from ROC and CART analysis reveals that CCL1 with a cut-off values of 21 pg/ml exhibits high levels of accuracy for the detection of active TB disease. Our findings clearly report that CCL1 is a stand-alone biomarker with ability to discriminate confirmed or unconfirmed TB from unlikely TB. In addition, CCL1 in combination of CXCL10 or CXCL1 will offer added advantage by increasing the sensitivity and specificity of the biomarker assays for further diagnosis. CCL1 will be further validated in a different endemic cohort for the development of a POC test, that can potentially be used as a future immunodiagnostic assay for pediatric TB. Our study also demonstrates that these chemokine biomarkers decrease in plasma levels following anti-TB treatment, confirming an association of these biomarkers with TB disease in children. In addition, our findings also revealed that at end of ATT in the confirmed TB group, most of the chemokine levels are still increased compared to the unlikely TB group indicating that the chemokines may take longer than six months to attain the normal range.

The strengths of this study involve the use of well characterised participant groups and, critically, the inclusion of an unlikely TB group, enabling us to determine the high sensitivity and specificity of the chemokine responses between the confirmed TB/unconfirmed TB vs unlikely TB. Some of the previous studies have examined the chemokine response in antigen-stimulated samples from the pediatric TB population. However, the drawback of using antigen-stimulated samples is the inconvenience in translating the assays to a point-of-care mode. Limitations of our study include a limited sample size, the absence of a validation cohort and the lack of inclusion of healthy control children. However, our results are promising enough to prompt further investigation of these biomarkers in large scale studies using diverse endemic populations. Hence, our study fills in the knowledge gap by performing profiling of plasma chemokines in unstimulated and stored plasma samples. Our study thus provides novel evidence for the utility of chemokines as biomarkers for pediatric TB.

## Supplementary Information

**Additional file 1: Figure S1.** The plasma levels of CCL1, CCL2, CCL3, CCL4, CCL11, CXCL1, CXCL2, CXCL9, CXCL10 and CXCL11 were measured in confirmed TB individuals at 6 months of anti-TB treatment (post-T) (n=24) and unlikely TB (n=76) individuals at baseline. The data are represented as scatter plots with each circle representing a single individual. P values were calculated using the Mann-Whitney test.

## Data Availability

The datasets used and/or analysed during the current study are available from the corresponding author on reasonable request.

## Abbreviations

ATT: Anti-tuberculosis therapy
CART: Classification and regression trees
GM: Geometric mean
*M.tb*: *Mycobacterium tuberculosis*
POC: Point-of-care
ROC: Receiver operator characteristics
TST: Tuberculin skin
TB: Tuberculosis
WHO: World Health Organization
