# Supplementary material for: Plasma chemokines as immune biomarkers for diagnosis of pediatric tuberculosis
Source: BMC Infect Dis. 2021 Oct 11;21:1055. doi: 10.1186/s12879-021-06749-6 (PMC8504024; doi:10.1186/s12879-021-06749-6)
Supplement: Supplementary file 1 — Additional file 1: Figure S1. The plasma levels of CCL1, CCL2, CCL3, CCL4, CCL11, CXCL1, CXCL2, CXCL9, CXCL10 and CXCL11 were measured in confirmed TB individuals at 6 months of anti-TB treatment (post-T) (n=24) and unlikely TB (n=76) individuals at baseline. The data are represented as scatter plots with each circle representing a single individual. P values were calculated using the Mann-Whitney test. [file 12879_2021_6749_MOESM1_ESM.pdf]

Figure S1

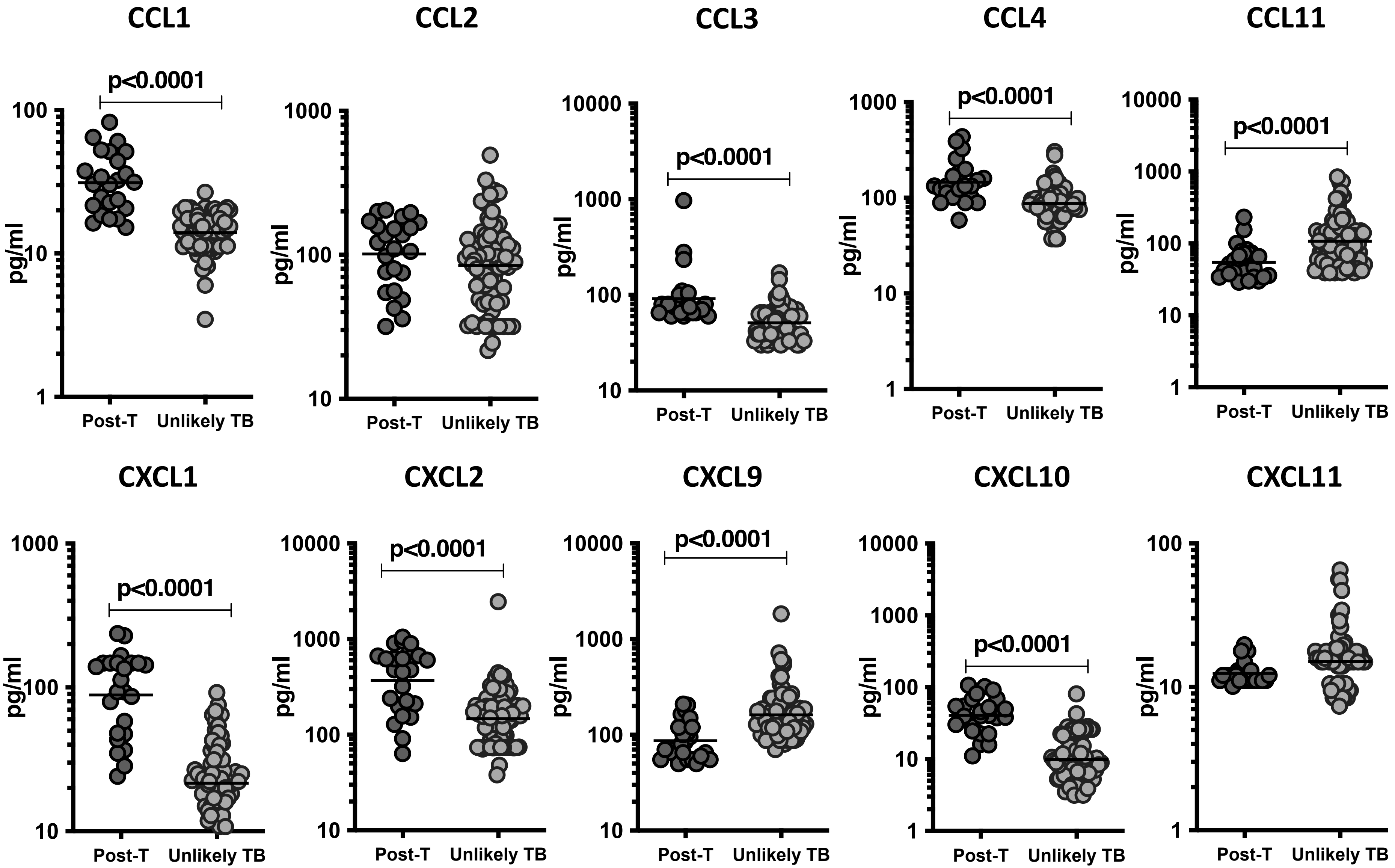

Figure S1: The plasma levels of CCL1, CCL2, CCL3, CCL4, CCL11, CXCL1, CXCL2, CXCL9, CXCL10 and CXCL11 were measured in confirmed TB individuals at 6 months of anti-TB treatment (post-T) (n=24) and unlikely TB (n=76) individuals at baseline. The data are represented as scatter plots with each circle representing a single individual. P values were calculated using the Mann-Whitney test.
